# A complex interplay between H2A.Z and HP1 isoforms regulates pericentric heterochromatin

**DOI:** 10.3389/fcell.2023.1293122

**Published:** 2023-11-09

**Authors:** Jessica González, Laia Bosch-Presegué, Anna Marazuela-Duque, Anna Guitart-Solanes, María Espinosa-Alcantud, Agustín F. Fernandez, Jeremy P. Brown, Juan Ausió, Berta N. Vazquez, Prim B. Singh, Mario F. Fraga, Alejandro Vaquero

**Affiliations:** ^1^ Chromatin Biology Laboratory, Josep Carreras Leukaemia Research Institute (IJC), Barcelona, Spain; ^2^ Tissue Repair and Regeneration Laboratory (TR2Lab), Institut de Recerca I Innovació en Ciències de La Vida i de La Salut a La Catalunya Central (IrisCC), Barcelona, Spain; ^3^ Nanomaterials and Nanotechnology Research Center (CINN), Spanish National Research Council (CSIC), El Entrego, Spain; ^4^ Institute of Oncology of Asturias (IUOPA), University of Oviedo, Oviedo, Spain; ^5^ Health Research Institute of the Principality of Asturias (ISPA), Oviedo, Spain; ^6^ Spanish Biomedical Research Network in Rare Diseases (CIBERER), Madrid, Spain; ^7^ Department of Immunology and Inflammation, Imperial College London, Commonwealth Building, The Hammersmith Hospital, London, United Kingdom; ^8^ Department of Biochemistry and Microbiology, University of Victoria, Victoria, BC, Canada; ^9^ Cytology and Histology Unit. Department of Cell Biology, Physiology, and Immunology, Universitat Autònoma de Barcelona (UAB), Barcelona, Spain; ^10^ Nazarbayev University School of Medicine, Astana, Kazakhstan

**Keywords:** HP1α,β,γ, heterochromatin, H2A.Z, epigenetics, genome stability, H3K9me3, H4K20me3

## Abstract

Pericentric heterochromatin (PCH) plays an essential role in the maintenance of genome integrity and alterations in PCH have been linked to cancer and aging. HP1 α, β, and γ, are hallmarks of constitutive heterochromatin that are thought to promote PCH structure through binding to heterochromatin-specific histone modifications and interaction with a wide range of factors. Among the less understood components of PCH is the histone H2A variant H2A.Z, whose role in the organization and maintenance of PCH is poorly defined. Here we show that there is a complex interplay between H2A.Z and HP1 isoforms in PCH. While the loss of HP1α results in the accumulation of H2A.Z.1 in PCH, which is associated with a significant decrease in its mobile fraction, H2A.Z.1 binds preferentially to HP1β in these regions. Of note, H2A.Z.1 downregulation results in increased heterochromatinization and instability of PCH, reflected by accumulation of the major epigenetic hallmarks of heterochromatin in these regions and increased frequency of chromosome aberrations related to centromeric/pericentromeric defects. Our studies support a role for H2A.Z in genome stability and unveil a key role of H2A.Z in the regulation of heterochromatin-specific epigenetic modifications through a complex interplay with the HP1 isoforms.

## Introduction

Proper organization of PCH is necessary for genome integrity. PCH plays a crucial role in the regulation of chromosomal architecture and centromere function. Alterations in PCH have been linked to chromosome segregation defects resulting in aneuploidy, muscular dystrophy, accelerated aging, and cancer (Peters et al., 2001; Hahn et al., 2010; Fioriniello et al., 2020). In mammals, PCH is composed of repetitive sequences, including major satellites in mice and α-satellites in primates (Guenatri et al., 2004; Thakur et al., 2021). Among the major hallmarks of PCH are two epigenetic modifications, namely, trimethylation of lysine 9 in histone H3 (H3K9me3) and trimethylation of lysine 20 in histone H4 (H4K20me3) (Peters et al., 2001; Jorgensen et al., 2013). H3K9me3 is catalyzed by the histone methyltransferase (HMTase) Suv39h1 that is a conserved regulator of heterochromatin structure and gene silencing in organisms ranging from humans to fission yeast (Nakayama et al., 2001; Peters et al., 2001; Schotta et al., 2002). H4K20me3 is catalyzed by Suv420h2 HMTase, requires H3K9me3 for its establishment and plays an important role in the regulation of cohesin deposition, chromatin compaction, DNA replication and cell cycle control (Hahn et al., 2013; Jorgensen et al., 2013). Another hallmark of PCH are HP1 proteins, structural proteins that exhibit high levels of sequence identity and differ mainly in a central hinge region (HR) that connects the N-terminal chromodomain with the C-terminal chromoshadow domain (Wang et al., 2000; Schoelz and Riddle, 2022). HP1 proteins can modulate the deposition and distribution of H3K9me3 and H4K20me3 (Lachner and Jenuwein, 2002; Hahn et al., 2013). Mammalian HP1 isoforms (Hp1α, β and γ) bind both H3K9me3 and Suv39h1 (Bannister et al., 2001; Lachner et al., 2001). This has led to a model where HP1 proteins act as regulators of H3K9me3 deposition and spreading through heterochromatin domains (Felsenfeld and Groudine, 2003). HP1 proteins also bind, to a lesser or greater extent, Suv420h2 HMTase, which provides a mechanism by which the recognition of H3K9me3 by HP1 proteins can recruit the H4K20me3 HMTase and promote the deposition of this mark in PCH regions (Hahn et al., 2013; Bosch-Presegue et al., 2017).

Although mammalian HP1 isoforms perform largely redundant roles in heterochromatin, reports from our group and others suggested that they have isotype-specific roles in the organization and the epigenetic regulation of heterochromatin structure (Dialynas et al., 2007; Bosch-Presegue et al., 2017). HP1α plays a role as an organizer of PCH structure together with CTCF, as its loss results in the accumulation of H4K20me3 and H3K27me3, as well as decreased accessibility and increased compaction in PCH regions (Bosch-Presegue et al., 2017). Loss of HP1α or HP1β have opposite roles in the distribution of H4K20me3 in PCH (Bosch-Presegue et al., 2017). For example, in contrast to HP1α, HP1β typically co-localizes with H4K20me3 in PCH, owing to both its preferential binding to Suv420h2, thereby targeting H4K20me3 to PCH foci, and its ability to bind specifically to H4K20me3 compared to HP1α and γ (Bosch-Presegue et al., 2017). Consistently, loss of HP1β results in PCH decompaction, whilst HP1α has the opposite effect (Bosch-Presegue et al., 2017).

The histone H2A variant H2A.Z is a key regulator of gene expression, chromatin organization and genome stability (Giaimo et al., 2019; Colino-Sanguino et al., 2022). It has two major isoforms, H2A.Z.1 and H2A.Z.2 that differ in just three amino acids and are encoded by two different genes (Dryhurst et al., 2009). H2A.Z.1 is the most widely expressed isoform and is essential in *Tetrahymena* and in all Metazoans investigated (van Daal and Elgin, 1992; Liu et al., 1996; Faast et al., 2001; Dijkwel and Tremethick, 2022). The role of H2A.Z in transcription is complex due to the interplay between the H2A.Z.1 and H2A.Z.2 isoforms with shared and unique isoform-specific interactors that include histone post-translational modifications (Sevilla and Binda, 2014; Corujo and Buschbeck, 2018; Kreienbaum et al., 2022). It is currently accepted that H2A.Z binds to active promoters and plays common and isoform-specific roles in RNA-polymerase II pausing/elongation (Giaimo et al., 2019). H2A.Z.1 and H2A.Z.2 regulate common and isoform-specific sets of genes (Dunn et al., 2017; Giaimo et al., 2019; Lamaa et al., 2020; Sales-Gil et al., 2021). H2A.Z isoforms are thought to be involved in different phases of the cell cycle, where H2A.Z.2 is proposed to regulate G_2_/M-associated genes, while H2A.Z.1 is thought to control G_1_/S phases *via* an interaction with c-Myc (Sales-Gil et al., 2021). H2A.Z.2 has also been specifically linked to DNA repair and to localize to sites of DNA damage (Boyarchuk et al., 2014; Fukuto et al., 2018).

The involvement of H2A.Z in the organization of PCH is complex and not well understood. It is reported that H2A.Z isoforms localize to PCH in *Drosophila* and mammals but not in plants (Rangasamy et al., 2003; Rangasamy et al., 2004; Greaves et al., 2007; Zilberman et al., 2008). Both mammalian H2A.Z.1 and H2A.Z.2 isoforms have been linked to the regulation of centromeric functions and chromosome segregation, although only H2A.Z.1 has been demonstrated to be physically associated to centromeres and pericentric heterochromatin in mammals (Rangasamy et al., 2003; Rangasamy et al., 2004; Greaves et al., 2007; Sales-Gil et al., 2021). Of note, loss of H3K9me3 or DNA methylation promote H2A.Z enrichment in PCH in mammals (Boyarchuk et al., 2014; Saksouk et al., 2014) while the single *Drosophila* H2A.Z ortholog H2Av is involved in the establishment of PCH structure and in the deposition of H3K9me2/3 (Swaminathan et al., 2005). In budding yeast there is evidence that H2A.Z acts as a regulator of chromatin boundaries, which indicates a role for this histone variant in the control of “spreading” of PCH (Meneghini et al., 2003). Notably, several studies have demonstrated an direct interplay between H2A.Z.1 and HP1α. H2A.Z.1 downregulation results in a decrease of HP1α enrichment in the chromosome arms (Rangasamy et al., 2004), which is consistent with the observation that H2A.Z.1 expression increases the binding of HP1α to the H3K9me3-marked chromatin fibers (Fan et al., 2004; Ryan and Tremethick, 2018). It is not known whether this interplay also operates in PCH regions and if H2A.Z.1 is associated to the other HP1 isoforms.

Here we describe for the first time, a molecular link between H2A.Z.1 and HP1β, rather than HP1α, in PCH. Our findings also indicate a specific role for H2A.Z.1 in regulating the deposition of heterochromatin epigenetic hallmarks within PCH that likely maintain the fidelity of chromosome segregation and genome stability.

## Materials and methods

### Cell culture studies

NIH3T3 and HEK293F cells were cultured in Dulbecco’s Modified Eagle medium (DMEM) (GIBCO, Invitrogen, Carlsbad, CA, United States) supplemented with 10% fetal bovine serum (GIBCO). *Wt*, *Hp1α* ^*−/−*^, *Hp1β* ^*−/−*^ and *Hp1γ* ^*−/−*^ mouse embryonic fibroblasts (MEFs) were generated and cultured in DMEM supplemented with 10% fetal bovine serum, Pen-strep (100U/mL), non-essential amino acids (GIBCO) and sodium pyruvate (Sigma S8636) according to the manufacturer’s instructions. noKO and reKO MEFs were generated from *Hp1α^−/−^* (KO) MEFs as previously described (Bosch-Presegué et al., 2017). All the cells were grown at 37°C in an atmosphere containing 5% CO_2_ and 90% humidity. In plasmid transfection experiments cells were transiently transfected using 3 µL of polyethylenimene at 1 mg/mL concentration (Polysciences Inc. 23,966) per µg of DNA. pcDNA4T0-HP1α/β/γ-RFP, pcDNA4T0-HP1α/β/γ-HA and pLVX-H2A.Z-EGFP were generated in pcDNA4T0 (Invitrogen) by standard PCR-based cloning strategy. pcDNA4T0-HP1β(α)-HA was generated by exchanging the hinge region (HR) from HP1β (aa79-113) to HP1α (aa78-117).

siRNA transfection experiments were performed with Lipofectamine 3,000 Reagent (Invitrogen L3000-001) using the manufacturer’s instructions, and 150 nM of the following siRNAs: siRNA universal Negative Control#1 (MISSION Millipore S1-001) and siRNA H2AFZ Mouse (siH2A.Z.1) (Dharmacon M-042994-01-0005).

### RNA isolation, cDNA synthesis, and RT-qPCR

Total RNA was purified using Maxwell RSC simplyRNA Tissue kit (Promega, AS1340). The cDNA was synthesized from 3 μg of total RNA with a Transcriptor First Strand cDNA Synthesis Kit (Roche) according to the manufacturer’s instructions. Real-time quantitative polymerase chain reaction (RT-qPCR) was performed using the QuantStudio 5 Real-Time PCR System (Thermo Fisher Scientific) with SYBR Green PCR Master Mix of Applied Biosystems. Relative gene expression was analyzed in QuantStudio 5 software (Thermo Fisher Scientific), and values were normalized to the expression of EEF2 and HPRT1. Details of oligonucleotides are shown below.

**Table:** 

	Forward (5'- 3′)	Reverse (5'- 3′)
*H2A.Z.1*	TAA​GGC​TGG​AAA​GGA​CTC​CGG​A	TCC​GTG​GCT​GGT​TGT​CCT​AGA​T
*H2A.Z.2*	GAT​CTC​AAA​GTG​AAG​CGC​ATC-	ATC​AGA​GAC​TTG​TGG​ATG​TGC​GGG
*EEF2*	TGT​CAG​TCA​TCG​CCC​ATG​TG	CAT​CCT​TGC​GAG​TGT​CAG​TGA
*HPRT1*	TCA​GTC​AAC​GGG​GGA​CAT​AAA	GGG​GCT​GTA​CTG​CTT​AAC​CAG

### ChIPs and reChiPs

ChIPs were performed with 3-5x10^6^ cells as previously described (Rodríguez-Ubreva and Ballestar, 2014). Cells were crosslinked with 1% methanol free-formaldehyde (PFA) and chromatin was sheared by sonication using Covaris M220 to an average fragment size of 250–750 bp. 60µg of sheared chromatin were used for each ChIP and were incubated ON with the following antibodies: 7.5 µg α-H2A.Z (Abcam, ab4174), 7.5 µg α-H2A (Cell Signaling, D603A) and 7.5 µg α-HA (Sigma-Aldrich, H6908), corresponding rabbit IgG (Diagenode, Liege, Belgium) was used as a control. Then, antibodies were conjugated to 30 µL protein A/G magnetic beads (Pierce, 26,262) to recover specific bounded chromatin and purified using NucleoSpin Gel and PCR Clean-Up (Macherey-Nagel, 740609250) and NTB buffer. RT-qPCR was conducted with SYBR Green PCR Master Mix (Applied Biosystems) according to the manufacturer’s instructions using the QuantStudio 5 Real-Time PCR System (Thermo Fisher Scientific). Primers used were: Major Satellites Fwd 5′-TGG​AAT​ATG​GCG​AGA​AAA​CTG-3′ and Rev 5′-AGG​TCC​TTC​AGT​GGG​CAT​TT-3’. Analysis was performed using the Percent Input method, and data was represented as ratio of the enrichment fraction with respect to input.

In re-ChIP experiments, the first ChIP (H2A.Z or H2A) was eluted with 10 mM Tris-EDTA and 20 mM DTT and diluted 20 times in dilution buffer (0.01% SDS, 1.1% Triton X-100, 1.2 mM EDTA, 16.7 mM Tris-HCl 8.1, 167 mM NaCl, and protease inhibitors) and proceeded to the second ChIP (HA).

siRNA H2A.Z.1 ChIPs were performed using LowCell ChIP kit™ protein A (Diagenode, C01010072) according the manufacturer’s instructions. The antibodies used in the ChIP were the following: 4µg/100.000 cells of α-H4K20me3 (Abcam, ab9053), 3µg/100.000 cells of α-H3K27me3 (Cell Signaling, 9,733), 2µg/100.000 cells of α-H3K9me3 (Abcam, ab8898) and 2µg/100.000 cells of α-H3K4me3 (Abcam, ab8580).

### Immunofluorescence and chromosomal aberrations studies

Immunofluorescence was carried out as described before (Serrano et al., 2013). Cells were fixed with 4% PFA for 7 min at RT and washed 3 times with PBS. Cells where further permeabilized with Buffer B (3% BSA, 0.2% triton PBS) for 10 min in agitation and incubated with Blocking buffer (3% BSA PBS) ON at 4°C in agitation for protein blocking. Primary antibody was added the following morning: α-H2A.Z (Cell Signaling, 2,718) using a 1:200 dilution in Buffer B for 1 h. After 3 washes with PBS, secondary α-rabbit 555 (Alexa Fluor, A21428) was added to cells at 1:1,000 dilution and incubated for 45 min. Cells were counterstained with DAPI solution (1 μg/mL in H_2_0) during 4 min, washed with H_2_0 and mounted in Vectashield. Images were acquired in a Leica TCS SP5 Confocal microscope at ×63 magnification, using 0.2-0.5 µm z-stacks. ImageJ software was used for image analysis.

For the fluorescence analysis of H2A.Z distribution at PCH foci Z projections were created from the Z-stacks acquired and converted to RGB files. A linear ROI was drawn on DAPI foci and the intensity profile for all the channels was generated using the macro “RGB profiles tool” in Fiji (Schindelin et al., 2012).

Chromosomal aberrations studies were conducted using the immunofluorescence protocol and a primary antibody anti-αtubulin (Sigma Aldrich, DM1A) and anti-CREST at a 1:200 dilution. The secondary antibody used was anti-mouse 488 (Life technologies) at a 1:1,000 dilution. Images were acquired in a Leica Stellaris Confocal microscope at ×63 magnification.

### FRET and FRAP

Fluorescence Resonance Energy Transfer (FRET) assays were performed as previously described (Bosch-Presegué et al., 2017). Briefly, NIH3T3 were cotransfected with 10 µg C1-H2A.Z.1-EGFP (donor plasmid) and 2 µg pcDNA4T0-HP1α/β/γ-RFP (acceptor plasmid) for 48 h and then, FRET was measured by time-correlated singlephoton counting (TCSPC) with a Leica TCS SP5 confocal microscope equipped with a single-molecule detection platform and single-photon counting electronics (PicoHarp 300, PicoQuant GmbH) using a HCX PL APO lambda blue 63x NA 1.4 Oil Objetive. Fluorescence recovery after photobleaching (FRAP) experiments were conducted as described previously (Bosch-Presegué et al., 2011). *Wt* and *Hp1α^−/−^* MEFs were infected with pLVX-H2A.Z.1-EGFP and selected using Puromycin 2 μg/mL 48 h prior to imaging, cells were transfected with 5 ug of Hp1γ-RFP. The experiments were carried out using a Leica Stellaris Confocal Microscope equipped with an on-stage incubation chamber set to 37°C and 5% CO_2_. Bleaching was performed in a circular area of 2 µm diameter at PCH foci using the 488-nm line from a 30 mW Argon laser at 50% power intensity. Images were processed using Fiji and data were double normalized and plotted with GraphPad Prism (GraphPad Software, Boston, Massachusetts United States). The recovery curves were fit to a one-phase association non-linear regression equation to calculate the half-life value.

### Immunoprecipitations and western blot

Co-immunoprecipitations were carried out in HEK293F cells transfected with: C1-H2A.Z.1-EGFP, pcDNA4T0-HP1α/β/γ-HA and pcDNA4T0-HP1β(α)-HA. Nuclear and chromatin extracts were prepared according to the Dignam protocol (Dignam et al., 1983) and using Benzonase (Sigma-Aldrich) for 6 h at 4°C in agitation. The lysates were clarified by centrifugation, and supernatants were incubated with α-HA agarose beads (Sigma, A2095) at 4°C ON in a rotator. The beads were gently washed 3 times with BC100 (100mM KCl, 10 mM Tris pH 7.8, 0.5% EDTA, 10% glycerol, 0.1 Mm PMSF and 0.1 mM DTT) and three times with BC500 (500mM KCl, 10 mM Tris pH 7.8, 0.5% EDTA, 10% glycerol, 0.1 mM PMSF and 0.1 mM DTT). The affinity-purified protein complexes were eluted by acidification using a buffer containing 0.2M glycine pH 2.3.

For the Western blot experiments cellular pellets were resuspended in Buffer Laemmli 1X (60 mM Tris pH 6.8, 10% glycerol, 2% SDS and 0.01% Bromofenol blue) supplemented with β-mercaptoethanol and sonicated 30 s into UP50H Ultrasonic Processor (Biotech) to obtain whole-cell extracts. SDS-PAGE was followed by transference into nitrocellulose membranes (GE Healthcare). The following primary antibodies were used: α-H2A.Z (Abcam, ab4174), α-HP1α (Euromedex, 2HP-2G9), α-HP1β (Euromedex, 1MOD-1A9), α-HP1γ (Euromedex, 2MOD-1G6), α-CTCF (Abcam, ab70303), α-actin (Sigma, A5316), α-GFP (Millipore, MAB2510) and α-HA (Sigma, H6908) all at 1:1,000 dilution except for α-actin (1:5,000), and incubated 1 h in agitation. HRP-conjugated mouse and rabbit secondary antibodies were from Sigma. Membranes were developed by Amersham™ ECL™ Western blotting Detection Reagents (GE Healthcare, Illinois, United States) and signals were detected by iBright FL1000 (Thermo Scientific). Band intensities were quantified using Quantity One Software (Biorad).

### Bisulfite pyrosequencing

DNA methylation levels for major satellites were analyzed using bisulfite pyrosequencing. Bisulfite modification of DNA was performed with the EZ DNA methylation-gold kit (Zymo Research) following the manufacturer’s instructions. Sets of primers for PCR amplification and sequencing were designed using the specific software PyroMark assay design (version 2.0.01.15). PCR amplification, pyrosequencing, and quantification of methylation were carried out using PyroMark Q24 reagents, equipment, and software (Qiagen). The following primers were used in this analysis.

**Table:** 

	Forward (5'- 3′)	Reverse (5'- 3′)	Sequencing (5'- 3′)
*MajorSat*	GGA​ATA​TGG​TAA​GAA​AAT​TGA​AAA​TTA​TGG	ACA​TAT​TCC​AAA​TCC​TAC​AAT​ATA​CAT	AAT​TAT​GGA​AAA​TGA​GAA​ATA​TTT​A

### Statistical analysis

The represented values show means of at least three independent experiments (n ≥ 3) with error bars representing standard error of means (SEM) unless otherwise specified. Statistical analysis was performed using a multivariant ANOVA (immunofluorescence analysis) or two-tailed Student’s t-test (rest of analysis). Specific n of each quantification and *p* values are indicated in the corresponding figure legends.

## Results

### HP1α loss promotes H2A.Z enrichment in pericentric heterochromatin

**FIGURE 1:**
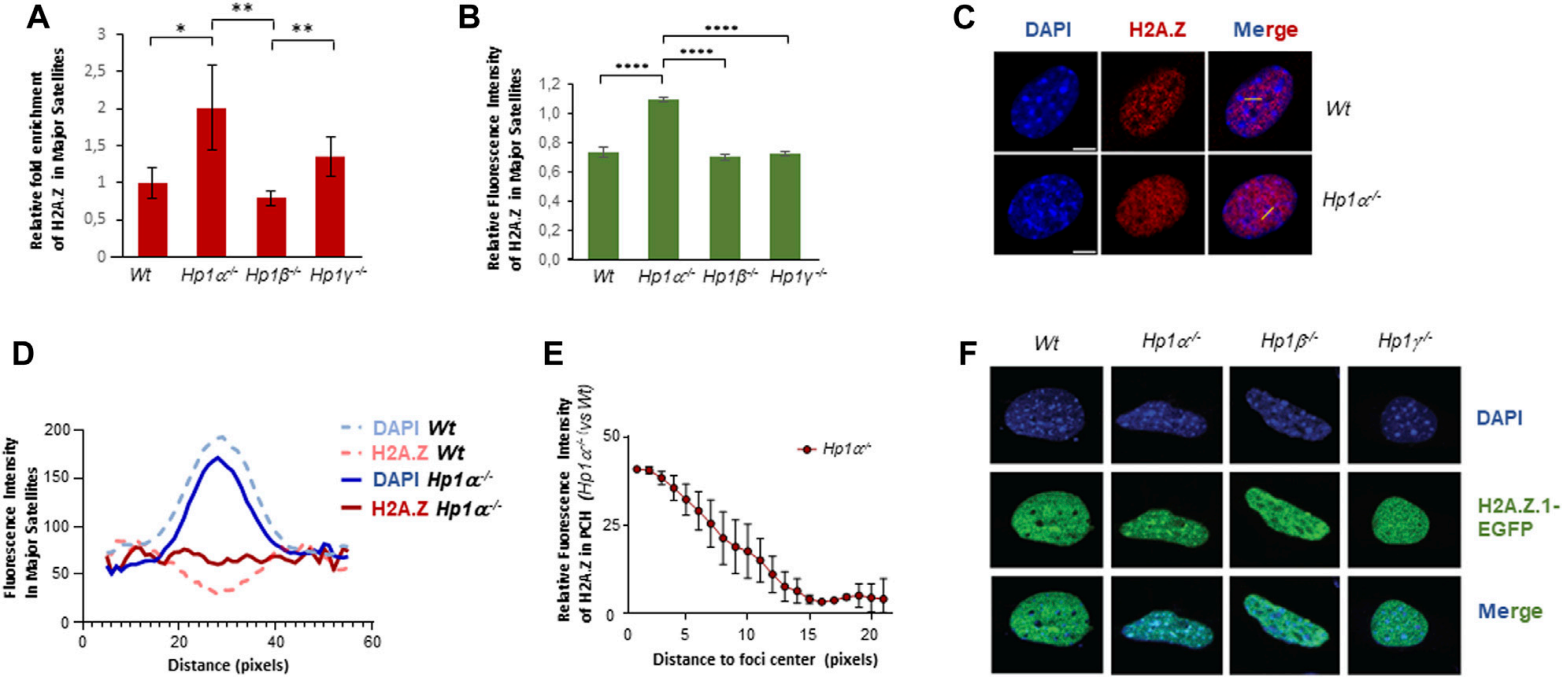
HP1α inhibits H2A.Z enrichment in PCH. **(A)** ChIP-qPCR of H2A.Z in major satellites of *Wt* or *Hp1α* ^*−/−*^, *Hp1β* ^*−/−*^ and *Hp1γ* ^*−/−*^ MEFs. A quantification of n = 4 independent experiments is shown. *p < 0.05, **p < 0.01, **(B)** Relative fluorescence intensity levels of H2A.Z in the PCH foci of the indicated MEFs, n = 20 cells analyzed for each condition. Representative images are shown in Supplementary Figure S1. ****p < 0.0001. **(C)** Immunofluorescence of endogenous H2A.Z in *Wt* and *Hp1α* ^*−/−*^ MEFs. Yellow lines indicate the regions where the fluorescent intensity was profiled. **(D)** Quantification of fluorescence intensity in linear densitometry analysis of IF experiments as in **(C)** showing H2A.Z distribution along sections of major satellites foci in *Wt* and *Hp1α* ^*−/−*^ MEFs. **(E)** Quantification of the relative increase of H2A.Z in the major satellite foci of *Hp1α* ^*−/−*^ MEFs compared to Wt, from the foci center to its periphery. Calculations were made by subtracting the mean fluorescence intensity of *Hp1α* ^*−/−*^ to Wt samples at each position of the linear selection within major satellites foci. **(F)** IF images of H2A.Z.1-GFP distribution in *Wt* or *Hp1α* ^*−/−*^, *Hp1β* ^*−/−*^ and *Hp1γ* ^*−/−*^ MEFs. Scale bar 5 μm.

Our aim in this work was to study the role of H2A.Z in PCH and its functional association to HP1 isoforms. We focused on H2A.Z.1 because it is the H2A.Z isoform that has been shown to localize to PCH (Greaves et al., 2007). ChIP experiments of H2A.Z showed a significant two-fold enrichment of this histone variant in cells lacking HP1α (Figure 1A). The levels of H2A.Z in HP1β-deficient cells did not change significantly while loss of HP1γ induced a mild enrichment (Figure 1A). We confirmed the specific effect of HP1α loss on H2A.Z in immunofluorescence studies (Figure 1B and Supplementary Figure S1). Linear densitometry analysis also showed that the DAPI-stained chromocenters in *Hp1α^−/−^* cells were smaller than those found in *Wt* cells and the accumulation of H2A.Z inversely correlated with DAPI intensity in these foci (Figures 1C, D). A more detailed analysis showed that the H2A.Z enrichment observed in the PCH foci of *Hp1α^−/−^* cells compared to *Wt* was at the center of the chromocenters (Figure 1E). We further confirmed the specific antagonistic interplay between HP1α and H2A.Z.1 in PCH through overexpression of H2A.Z.1-EGFP in *Wt*, *Hp1α^−/−^*, *Hp1β^−/−^* and *Hp1γ^−/−^* MEFs. These analyses showed a strong accumulation of H2A.Z.1 in PCH only in HP1α-deficient cells (Figure 1F), indicating that HP1α negatively regulates H2A.Z.1 enrichment in PCH, in contrast to work showing a linear relationship between HP1α and H2A.Z.1 (Fan et al., 2004; Ryan and Tremethick, 2018).

### HP1α regulates H2A.Z.1 dynamics in PCH

**FIGURE 2:**
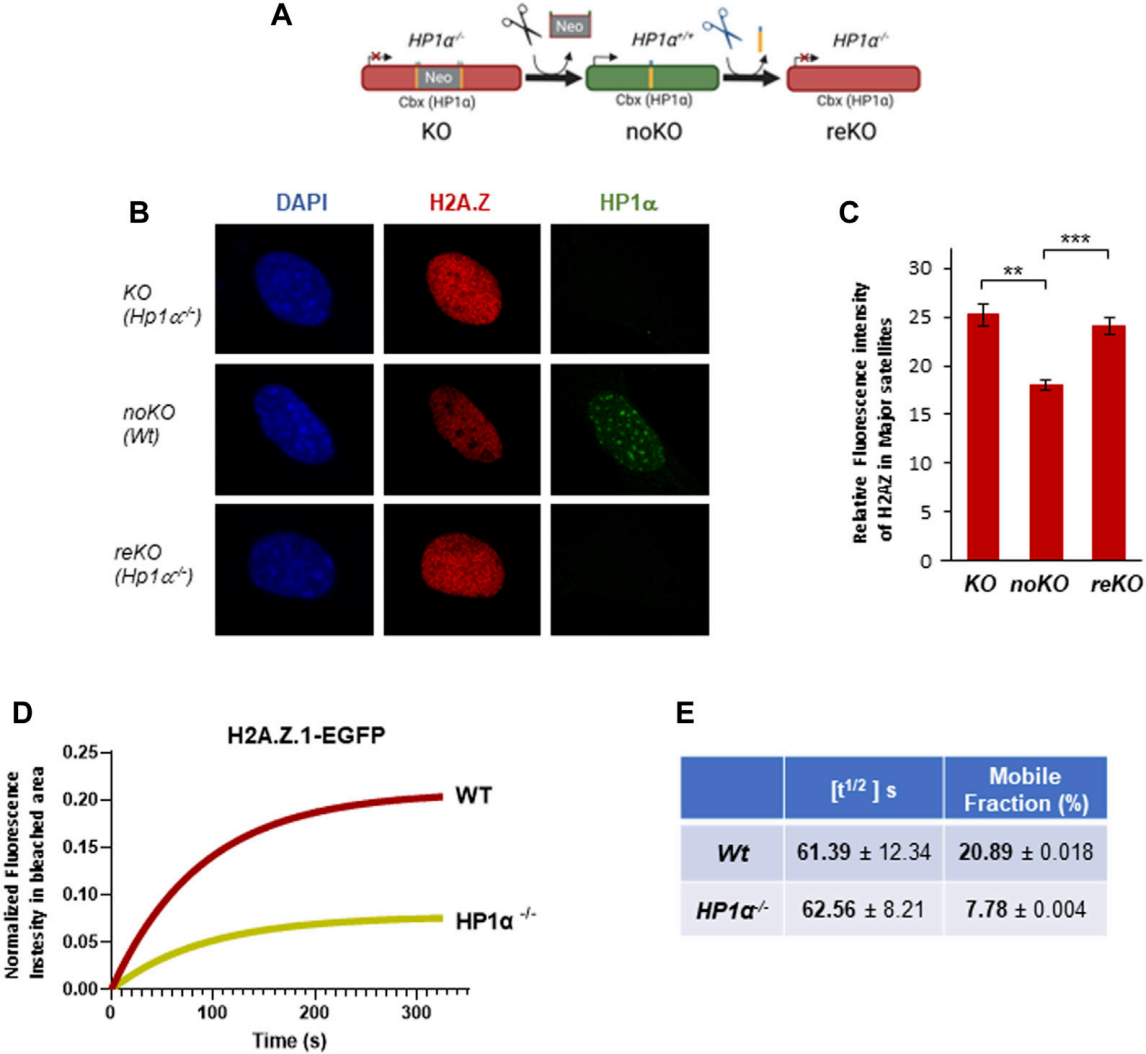
HP1 regulates H2A.Z.1 mobile fraction in PCH. **(A)** Schematic representation of noKO and reKO generation from *Hp1α* ^*−/−*^ MEFs (KO). **(B)** Representative IF images of H2A.Z and HP1α in the nucleus of KO (Hp1α^−/−^), noKO (Wt) and reKO (*Hp1α* ^*−/−*^) MEFs, **(C)** Relative fluorescence intensity levels of H2A.Z in the PCH foci of the indicated MEFs, n = 15 cells analyzed for each condition. **p < 0.01, ***p < 0.001. **(D)** Fluorescence intensity recovery of FRAP assays in PCH foci for H2A.Z.1-EGFP in *Wt* and *Hp1α* ^*−/−*^ MEFs. Intensity values were corrected for fluorescence fluctuations and double normalized before fitting the curves to a non-linear regression model. **(E)** Quantification of the FRAP analysis (n = 20 cells for each condition) measured parameters including half-time of fluorescence recovery (t^1/2^ (s)) and the mobile fraction (%).

Aiming to understand better the interplay between HP1α and H2A.Z.1, we took advantage of our engineered *Hp1α^−/−^* mice, where HP1α expression can be restored through the expression of CRE recombinase (termed noKO cells) and then again disrupted by expressing FLP recombinase (termed reKO) (Bosch-Presegue et al., 2017) (Figure 2A). We found significant decrease of H2A.Z in PCH in noKO cells (Figures 2B, C), which was reversed in reKO cells (Figures 2B, C). These results support an involvement of HP1α in H2A.Z.1 localization to PCH. To study the impact of HP1α in H2A.Z.1 dynamics, we performed FRAP analysis in *Wt* and *Hp1α^−/−^* MEFs expressing H2A.Z.1-EGFP, together with HP1γ-RFP to mark PCH foci in live cells. We confirmed that the expression of H2AZ.1-EGFP was significantly lower than endogenous H2A.Z (Supplementary Figure S2A). FRAP of PCH showed that in *Wt* cells H2A.Z.1 has a half-time residence [t^1/2^] of 61.39s with around 20% of H2A.Z.1 residing in the mobile fraction. Loss of HP1α did not alter H2A.Z [t^1/2^] but decreased the proportion of H2A.Z.1 mobile fraction in to 7.8% (a 60% decrease) in *Hp1α^−/−^* cells (Figures 2D, E and Supplementary Figure S2B).

### H2A.Z preferentially interacts with HP1β within PCH

**FIGURE 3:**
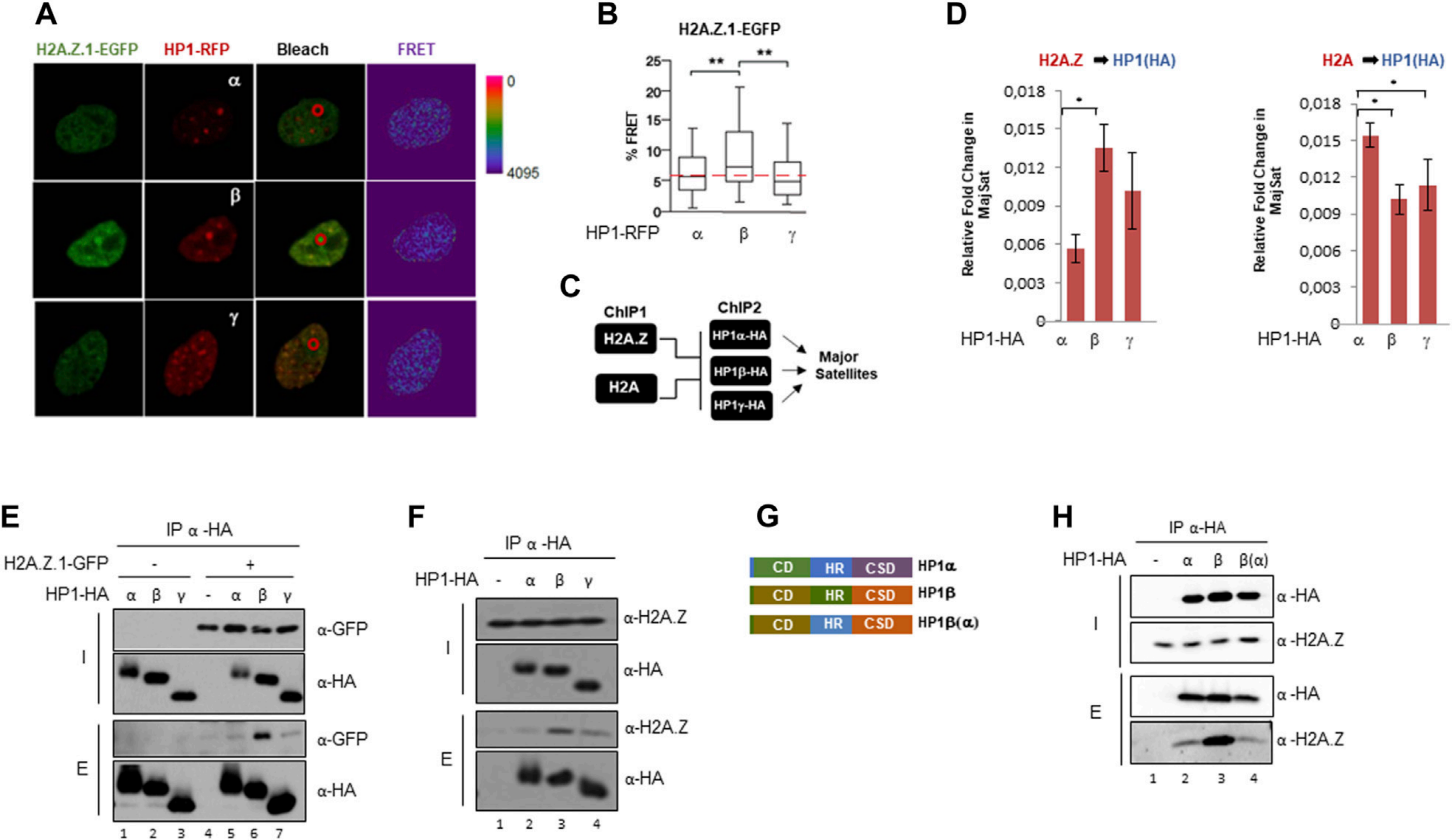
H2A.Z.1 preferentially interacts with HP1β in PCH regions. **(A)** Representative images of the FRET experiments between H2A.Z.1-EGFP and HP1-RFP in PCH foci of NIH3T3 cells. The bleaching area is marked with a red circle. **(B)** Relative quantification of FRET analysis showed in **(A)**. **p < 0.01. Relative FRET % was calculated considering 100% as the FRET value that was obtained for GFP-RPF (positive control) and 0% as the value obtained for the FRET value that was obtained for the donor construct alone. **(C)** Schematic representation of reChIPs experimental workflow. First ChIP was performed against H2A.Z or H2A followed by a second ChIP against HP1α-HA, HP1β-HA or HP1γ-HA. **(D)** ReChIPs experiments of endogenous H2A.Z or H2A (ChIP #1) and HP1-HA isoforms (ChIP #2) in major satellites of NIH3T3 cells previously transfected with HA-tagged HP1 isoforms. Relative fold enrichment for HP1(HA) isoforms in H2A.Z (left) or H2A (right) ChIP elutions in Major Satellites are indicated. A quantification of n = 3 independent experiments is shown. *p < 0.05. **(E)** HA Immunoprecipitation experiments in nuclear extracts of HEK293F expressing H2A.Z.1-GFP and the three HA-tagged HP1 isoforms. Inputs (I) and elutions **(E)** are shown, **(F)** HA Immunoprecipitation as in **(E)** to test interaction between endogenous H2A.Z and HP1-HA isoforms in co-IP experiments. **(G)** Schematic domains representation of HP1α, HP1β and HP1β(α) generated by exchanging the hinge region (HR) from HP1β (aa79-113) to HP1α (aa78-117) (Raurell-Vila et al., 2017). **(H)** Interaction between endogenous H2A.Z and HP1α, HP1β and HP1β(α)-HA in co-IP experiments using HA beads in whole cell extracts of HEK293F cells.

We next investigated whether the interplay between H2A.Z and HP1 isoforms within the PCH takes place *in vivo*. Accordingly, we performed FRET assays, where we analyzed the *in vivo* binding between H2A.Z.1-EGFP and HP1α/β/γ-RFP (Figures 3A, B). The results showed a greater interaction between H2A.Z.1 and HP1β in PCH regions compared to HP1α or γ. This preferential binding was supported by re-ChIP studies in cells expressing HA-tagged HP1 α, β and γ. (Figures 3C, D). We used HA-tagged HP1 isoforms to overcome the observed differences in efficacy of the isoform-specific antibodies. For re-ChIP a first round of ChIP was performed using either H2A.Z or H2A.The elutions were then used as input for a second round of ChIPs against HA-tagged HP1α, β or γ (Figure 3C). Confirming the FRET results, the association of HP1β and H2A.Z with major satellite sequences in PCH was greater compared to the other HP1 isoforms. HP1α tended to colocalize more with H2A (Figure 3D). In contrast to HP1α or HP1β, HP1γ did not show any preferential association with H2A.Z or H2A (Figure 3D). The HP1 isoform-specific relationship between HP1s and H2A.Z.1 was further supported by immunoprecipitation experiments. We observed significant preference between overexpressed or endogenous H2A.Z.1 with HP1β (Figures 3E, F). The main difference between HP1α and HP1β is in the hinge region (HR) that connects the N-terminal chromodomain (CD) with the C-terminal chromoshadow domain (CSD) (Figure 3G). We next replaced the HP1β HR with the HP1α HR, resulting in the chimeric HP1 protein we termed HP1β(α) (Figure 3G) and studied the interaction between HP1β(α) and H2AZ.1 compared to HP1β and HP1α. The results showed that the HP1β(α) chimera had a decreased ability to interact with H2A.Z.1 (Figure 3H), suggesting that the HP1β HR can regulate the interaction between HP1β with H2A.Z.1.

Altogether, our results suggest a direct, specific, interaction between HP1β and H2A.Z.1 in PCH regions that is regulated by HP1β HR.

### H2A.Z.1 loss promotes hyper heterochromatinization of PCH and results in increased genome instability related to centromeric defects

**FIGURE 4:**
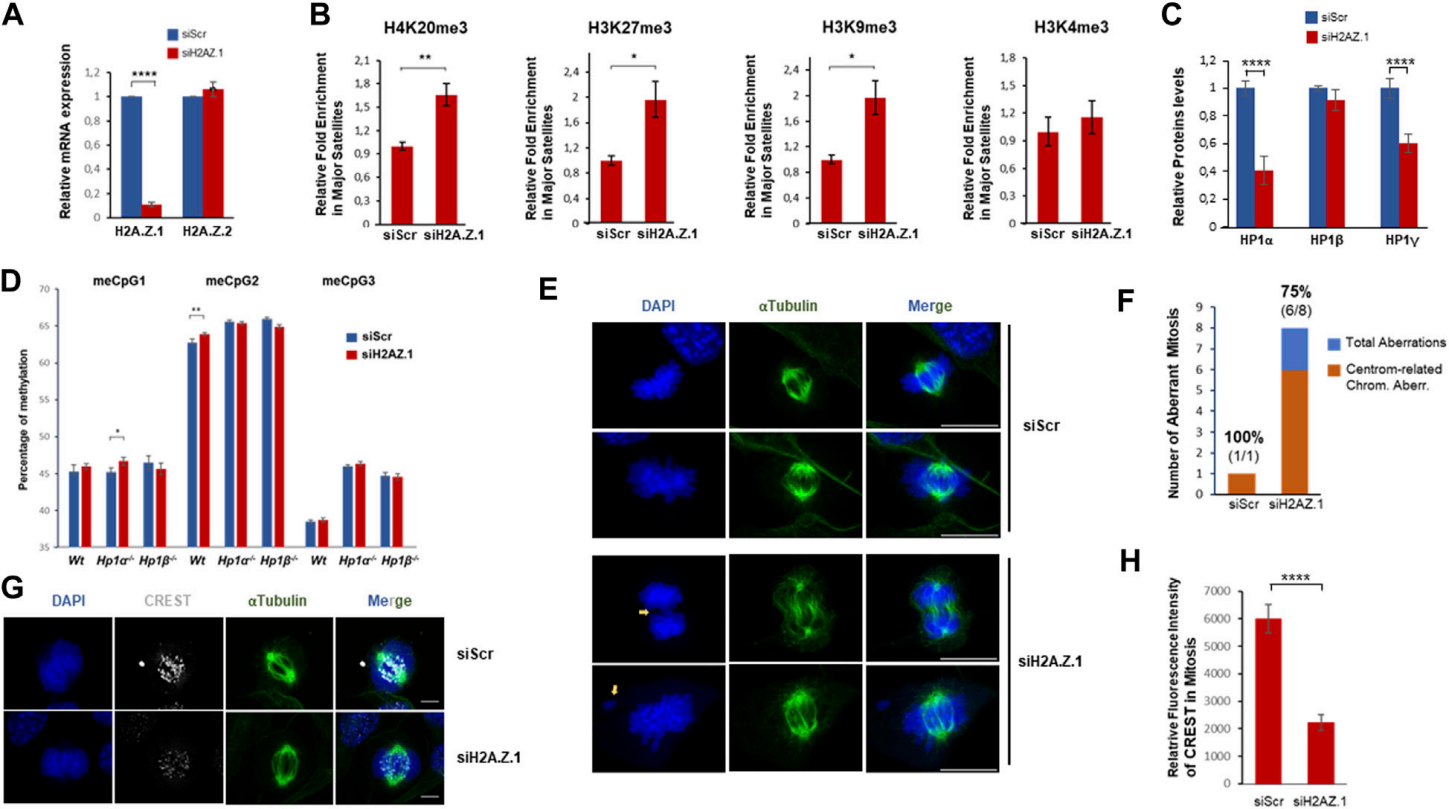
H2A.Z.1 downregulation induces hyper heterochromatinization of PCH regions and increased levels of chromosomal aberrations linked to segregation defects. **(A)** RNA expression levels of H2A.Z.1 and H2A.Z.2 upon expression of siScramble (siScr), siH2A.Z.1 or siH2A.Z.2 NIH3T3 cells. **(B)** ChIP-qPCR of H4K20me3, H3K27me3, H3K9me3 and H3K4me3 in major satellites of siRNA Scr and siRNA H2A.Z.1 NIH3T3 cells. A quantification of n = 4 independent experiments is shown. *p < 0.05, **p < 0.01. **(C)** Quantification of western-blots of siScr and siH2A.Z.1 NIH3T3 cells analyzed in **(A,B)** to monitor HP1 protein levels in whole-cell extracts normalized by Actin levels. A representative image is shown in Supplementary Figure S3. **(D)** DNA methylation levels of three representative meCpG (#1-3) in PCH. Bisulfite pyrosequencing was performed in MEFs *Wt*, *HP1α* ^*−/−*^ and *HP1β* ^*−/−*^ upon siRNA-driven downregulation or not of H2A.Z.1. A relative quantification (n = 4) of siH2A.Z.1 vs. siScramble (Scr) in each case is shown. *p < 0.05, **p < 0.01. **(E–G)** Chromosomal aberration analysis of these cells. **(E)** Representative IF images showing chromosomal aberrations in siRNA scr and siRNA H2A.Z.1 NIH3T3 cells. DAPI (blue) and α-tubulin (green) are shown. Scale bar 10 µm. **(F)** Quantification of the analysis in **(E)** of the frequency of observed chromosomal aberrations detected (blue) and the proportion of them that are associated to centromere and segregation defects (in orange; spell out “centromere-related chromosomal aberrations”). Above the bars, the frequency of aberrations associated to these centromere and segregation defects (in bold) and the detected number of aberrations (segregation aberrations/total number) are shown. **(G)** IF as **(F)** of mitotic cells staining with CREST antibodies (white). Scale bar 5 µm. **(H)** Quantification of the CREST fluorescence signal in the IF of **(G)** (siScr n = 15; siH2A.Z.1 n = 18). ****p < 0.001.

We next knocked-down H2A.Z.1 in NIH3T3 cells (Figure 4A) to study the contribution of H2A.Z.1 to pericentric heterochromatin structure. ChIP-qPCR showed that knockdown of H2A.Z.1 increased the levels of the three heterochromatic epigenetic hallmarks (H4K20me3, H3K27me3 and H3K9me3) at the major satellite sequences contained with PCH (Figure 4B). No change was observed in the active mark H3K4me3 (Figure 4B). These data indicate that H2A.Z.1 plays an important role in the regulation of the heterochromatic epigenetic marks that define PCH structure and therefore in PCH integrity. Notably, our previous work showed that loss of HP1α resulted in an enrichment of H4K20me3 and H3K27me3 (Bosch-Presegue et al., 2017). These previous findings together with our observation showing that H2A.Z.1 downregulation results in a global decrease in HP1α and HP1γ levels (Figure 4C) suggest that the increase in H4K20me3 and H3K27me3 may, at least in part, be owing to a decrease in HP1α levels (Figure 4C). Surprisingly, in contrast to the effect of H2A.Z.1 downregulation of these histone marks, we did not detect clear differences in the levels of DNA methylation within PCH in *Wt* MEFs, *Hp1α* ^*−/−*^ or *Hp1β* ^*−/−*^. However, we did observe that H2A.Z.1 loss resulted in a very mild but significant increase in any of the three meCpGs tested in the case of *Wt* or *Hp1α* ^*−/−*^ MEFs. In contrast, H2A.Z.1 downregulation in HP1β-deficient MEFs had an opposite albeit mild effect. These results indicate that the reported antagonism between H2A.Z and DNA methylation is not valid in PCH regions (Figure 4D) (Zilberman et al., 2008; Boyarchuk et al., 2014).

PCH plays a crucial role in chromosome segregation during mitosis so we were prompted to investigate the effect of H2A.Z.1 knockdown on segregation defects. We found that decreased levels of H2A.Z.1 resulted in a significant increase in mitotic chromosome abnormalities. The percentage of abnormal mitoses in H2A.Z.1 knockdown cells was 45% compared to 5.8% in *Wt* cells (Figures 4E, F). 75% of the abnormal mitoses in H2A.Z.1 knockdown cells were typical of defects associated with loss of centromere/pericentromere function, such as lagging chromosomes or chromosomes that were not attached to microtubules (Figures 4E, F) (Warecki and Sullivan, 2022). Immunofluorescence experiments of kinetochore proteins also revealed the presence of these phenotypes (Figure 4G), supporting a direct impact of H2A.Z.1 in chromosomal stability through a regulation of centromeric/pericentromeric regions.

## Discussion

**FIGURE 5:**
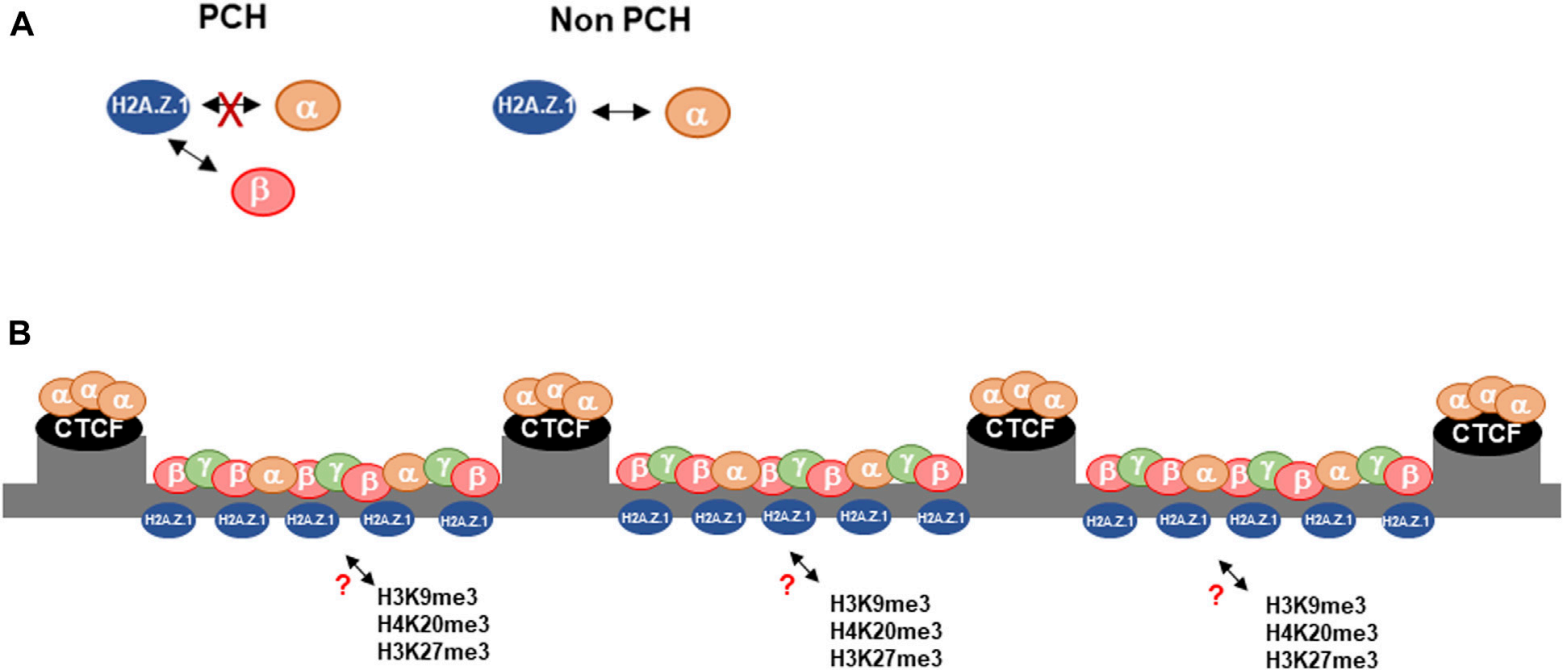
Proposed model of the interplay between H2A.Z.1 and HP1α/β in PCH. **(A)** In contrast to the rest of the genome, H2A.Z.1 and HP1α show an antagonistic interplay in PCH, where it interacts preferentially with HP1β. **(B)** We suggest that the role of HP1α as an organizer of PCH compartmentalization regulates the deposition of H2A.Z.1 through HP1β. In turn H2A.Z.1 controls the establishment of heterochromatin hallmarks such as H4K20me3, H3K9me3 and H3K27me3 through an undefined mechanism.

Previous studies have shown a functional relationship between H2A.Z.1 and HP1α (Fan et al., 2004; Rangasamy et al., 2004; Greaves et al., 2007; Ryan and Tremethick, 2018). In this work we show that the interplay between H2A.Z.1 and HP1α is complex and involves other HP1 isoforms (Figure 5). These interactions, are likely to play a role in the regulation and organization of PCH that will, in turn, have implications for how PCH maintains genomic stability. HP1α negatively regulates H2A.Z.1 deposition at PCH. In HP1α-deficient cells H2A.Z.1 accumulates at the PCH, which is associated with decreased fraction of mobile H2A.Z.1, but with little change in the residence times ([t^1/2^]) of bound H2A.Z.1. These data indicate that effect of HP1α depletion on H2A.Z.1 is indirect rather than owing to direct HP1α:H2A.Z.1 interaction. We observed that HP1α depletion results in smaller DAPI-stained chromocenters in *Hp1α^−/−^* cells, which is unexpected if the function of HP1α is to act as boundaries of that stop the “spreading” of PCH, as previously suggested (Bosch-Presegue et al., 2017). Although our evidence suggests that this interplay involves H2AZ.1 but not H2AZ.2, we cannot discard completely that part of these effects are not mediated also by H2AZ.2. The fact that these isoforms differ in only three amino acids, makes very difficult to distinguish them at molecular level unless they are specifically tagged. Future studies should clarify whether H2AZ.2 contributes in any way to the interplay we have identified.

An additional and complementary explanation that would accommodate an indirect effect of HP1α depletion on H2A.Z.1 deposition at PCH, is our finding of the direct interaction of H2A.Z.1 with HP1β. Recent studies in fission yeast have shown that H3K9me-marked chromatin is bound by HP1 proteins, Swi6 and Chp2, and form binding platforms that recruit HP1 binding partners resulting in macromolecular complexes that are predominantly in a chromatin-bound state (Chen et al., 2023). In our scenario HP1β bound to H3K9me3 and/or H4K20me3 could recruit H2A.Z.1, whilst HP1α that cannot bind as efficiently to H2A.Z could act indirectly by competing with HP1β for binding to H3K9me3 in PCH. The model is consistent with our observation that H2A.Z.1 accumulates in PCH in *Hp1α^−/−^* cells with a decrease in the H2A.Z.1 mobile fraction. Implicit in the model is that there is a fine balance in the levels and distribution of HP1 isoforms that regulate the stable recruitment of HP1-interacting proteins to PCH. However, two observations suggest that the competition model, as stated, may be too simple: First, considering that HP1 isoforms can homo- and heterodimerize a HP1α:HP1β dimer could bind to H3K9me3 and recruit at the same time H2A.Z to PCH(Canzio et al., 2014). Second, at protein levels evidence suggest that there is a compensatory effect between HP1β and γ, but not α, as loss of HP1 β results in a general increase in HP1γ protein levels but not HP1α, and *vice versa* (Raurell-Vila et al., 2017). Future studies should determine the functional relevance of this competition model.

The other major finding of this work is the general increase of heterochromatin hallmarks in PCH such as H3K9me3, H4K20me3 and H3K27me3 upon downregulation of H2A.Z.1. This suggests that H2A.Z.1 regulates deposition of these marks in PCH. The mechanism involved is not known, but may in part be explained by the decrease in HP1α protein levels, but not HP1β, in cell extracts from H2A.Z.1 depleted cells, which is consistent with previous reports whereof the downregulation of H2A.Z.1 leads to a loss of HP1α localization within chromosome arms (Rangasamy et al., 2004). As consequence of HP1α loss in H2A.Z.1 depleted cells would enhance binding of HP1β in PCH through the removal of the HP1α competitor. Increased HP1β binding would enhance the recruitment of HMTases that would, in turn, increase the levels of histone modifications H3K9me3, H4K20me3 and H3K27me3.

Previous FISH-DNA analysis of H2A.Z.1 siRNA knock-down in L929 fibroblasts showed that depletion of H2A.Z results in a gradual decompaction of the chromocenter (Greaves et al., 2007), which disagrees with our evidence. These discrepancies may be due to the use of different cell lines in different stages of differentiation (MEFs vs. L929), or the technical approach. Nevertheless, our previous evidence on PCH hypercompaction upon loss of HP1α, and its link to H2A.Z are in full agreement with our observations in this work.

Our findings on the increased levels of chromosomal aberrations are in fully agreement with previous reports that showed a role for H2A.Z in chromosome segregation and in the control of centromeric architecture and pericentric heterochromatin integrity (Rangasamy et al., 2004; Greaves et al., 2007; Sales-Gil et al., 2021). This evidence underscores the crucial role of H2A.Z in genome stability beyond the regulation of gene expression, which is gradually being understood. Considering the well-established link between H2A.Z isoforms and different types of cancer (Dryhurst et al., 2012; Vardabasso et al., 2015; Yang et al., 2016), further characterization of the role of H2A.Z isoforms in heterochromatin should provide valuable evidence to understand the molecular basis of the onset of some of these cancers.

## Data Availability

The original contributions presented in the study are included in the article/Supplementary material, further inquiries can be directed to the corresponding author.
