# Supplementary material for: A complex interplay between H2A.Z and HP1 isoforms regulates pericentric heterochromatin
Source: Front Cell Dev Biol. 2023 Nov 9;11:1293122. doi: 10.3389/fcell.2023.1293122 (PMC10665487; doi:10.3389/fcell.2023.1293122)
Supplement: Supplementary file 1 [file DataSheet1.PDF]

SUPPLEMENTARY INFORMATION

SUPPLEMENTARY FIGURES

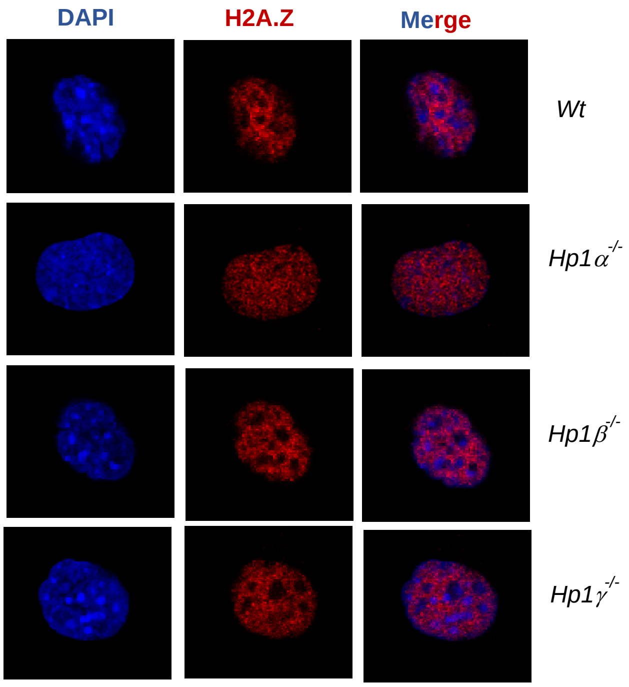

Supplementary Figure S1. Representative images of the immunofluorescence quantitative analysis shown in Figure 1B.

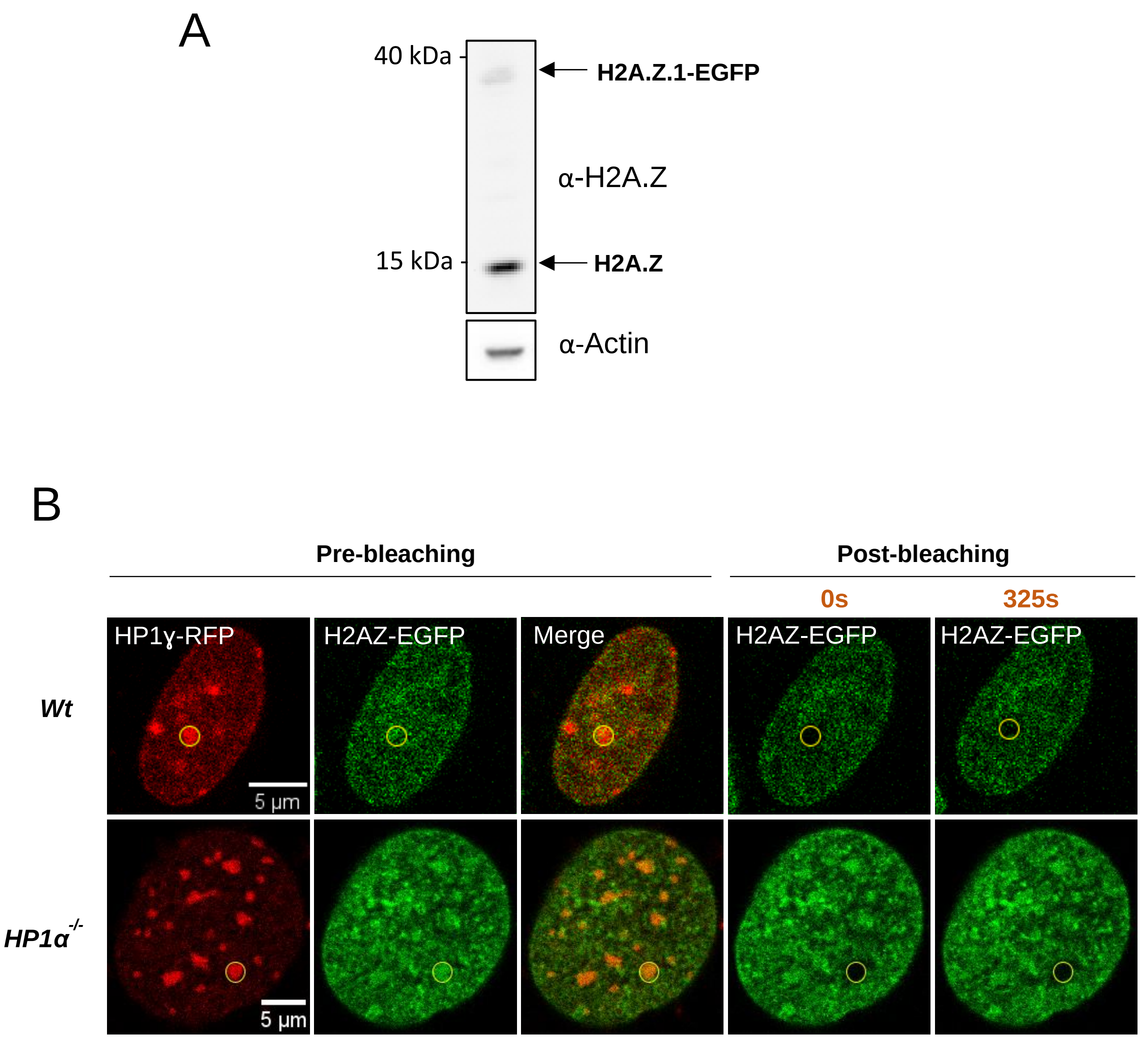

Supplementary Figure S2. **(A)** Western-blot of H2A.Z in *Wt* MEFs overexpressing H2A.Z.1-EGFP. **(B)** Representative pre and post-bleaching images of the FRAP analysis shown in Figure 2D-E. Indicated in yellow circle the bleached PCH foci marked by HP1$_{\gamma}$-RFP.

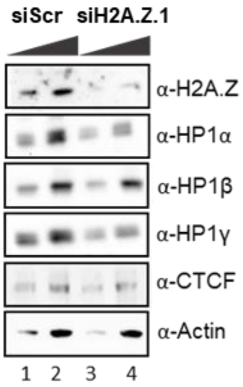

Supplementary Figure S3. Representative western-blot of n=3 quantified in Figure 4C. Black triangles indicate two increasing amounts of cell extract loaded (x and 3x).
